# Quiescence in *Aedes aegypti*: Interpopulation Differences Contribute to Population Dynamics and Vectorial Capacity

**DOI:** 10.3390/insects9030111

**Published:** 2018-09-01

**Authors:** Luciana O. Oliva, Roseli La Corte, Marcelo O. Santana, Cleide M. R. de Albuquerque

**Affiliations:** 1 Departamento de Zoologia, Centro de Biociências, Universidade Federal de Pernambuco (UFPE), Recife 50670-901, Brazil; 2 Departamento de Morfologia, Centro de Ciências Biológicas e da Saúde, Universidade Federal de Sergipe (UFS), São Cristóvão 49100-000, Brazil; rlacorte@ufs.br; 3 Departamento de Educação em Saúde, Universidade Federal de Sergipe (UFS), Lagarto 49400-000, Brazil; mcelooliva@gmail.com

**Keywords:** biological cycle, Culicidae, desiccation resistance, development, egg dormancy, fitness, mosquito, plasticity, reproduction

## Abstract

The strategy of *Aedes aegypti* to prolong embryonic viability by quiescence has severe implications for geographic expansion and maintenance of mosquito populations in areas under control measures. We evaluated the effects of quiescence on biological parameters directly or indirectly associated with population dynamics and vectorial capacity in populations of this mosquito species from two Brazilian municipalities characterized as dengue, chikungunya, and Zika transmission areas. Egg viability, initial hatching time, post-embryonic development time, adult emergence rate, sexual proportion, adult size, fecundity, and fertility were analyzed using eggs stored for 10, 40, 70, 100, 130, and 160 d. Quiescence time reduced overall egg viability and post-embryonic development time in both municipalities but was more costly in Aracaju (100 d, 8 d) than in Recife (130 d, 7.5 d). Emergence rates increased in Recife when the eggs were older, but not in Aracaju. Significant deviations in sexual proportion, with male predominance, were observed in both populations. Initial hatch, fecundity, fertility, and adult size did not significantly influence egg quiescence time. These results indicate intrinsic and differential characteristics for each *A. aegypti* population, suggesting a differential cost of quiescence for population dynamics parameters that can indirectly affect vectorial capacity and control measures.

## 1. Introduction

Arboviruses transmitted by mosquito vectors have been a major cause of global health problems, particularly in tropical and subtropical countries [1,2,3,4,5]. Viruses causing dengue fever, urban yellow fever, chikungunya fever, and Zika virus disease are some arboviruses whose main transmission vector in these areas is *Aedes aegypti* (L.) [6,7,8,9,10].

Over the course of its evolutionary history, *A. aegypti* has developed strategies favoring the explosive growth of natural populations, invasion, and dissemination worldwide [11,12,13,14,15,16]. One of these strategies is the capacity of eggs to resist desiccation, mainly due to low humidity and high temperatures, until conditions become favorable for hatching (non-seasonal quiescence) [17,18,19,20,21]. Resistant eggs can allow the pharate first instar larvae to survive inside the egg in unfavorable environments for up to over a year [12,22,23,24]. Thus, quiescent eggs constitute a significant problem for vector control because these eggs can directly contribute to the maintenance of mosquito populations in treated areas, and may facilitate the transportation of eggs and the establishment of new populations [22,25]. In subtropical regions, low annual average temperatures are a limiting factor for the survival of *A. aegypti*. However, in a current global warming scenario, the elevation of temperatures in these areas can favor the introduction, dispersion, and expansion of *A. aegypti* [26,27]. Furthermore, increases in temperature may reduce the incubation period of some pathogens, including dengue virus, shortening the dengue transmission cycle [28,29]. Considering the existence of trans-ovarian transmission in this vector [30,31], quiescent eggs may play a crucial role in this situation since eggs can remain viable in the environment for long periods of time.

Although scarcely studied, there are indications that the quiescence process has a negative cost on the fitness of *A. aegypti* individuals. For instance, the extension of egg quiescence periods has been shown to negatively affect larval physiology and development by reducing lipid reserves [32] and reducing female body masses and reproductive fitness [33]. Such observations raise a key question as to whether quiescence would shape parameters directly or indirectly associated to population dynamics, vector capacity, and competence (i.e., mosquito density, time of post-embryonic development, sexual proportion, fecundity, and fertility), and how different such effects would be on different mosquito populations. Moreover, previous studies have shown that local and regional genetic differences between mosquito populations can affect different characteristics of the biology of vectors [34,35]. Thus, a role for interpopulation variations in egg quiescence in population dynamics and vector capacity is a reasonable assumption, but this remains uninvestigated.

Using an approach that combines biological parameters capable of directly or indirectly affecting population dynamics and vector capacity we investigated: (1) the differences in the eclosion rate from quiescent eggs in distinct *A. aegypti* populations from transmission areas for dengue fever, chikungunya fever, and Zika; (2) whether the initial hatching time, post-embryonic development time, adult emergence rate and sexual proportion were differentially affected by the quiescence period in these populations; (3) whether fecundity, fertility, and adult size are altered as result of increased quiescence time. We tested the hypothesis that the duration of the quiescent period and its consequences on individual fitness are intrinsic characteristics of each population, differing between *A. aegypti* populations due to variations in genetic features and selective environmental pressures. Therefore, we predict that quiescence will negatively affect the initial hatching time by increasing the time required for larvae eclosion due to a greater necessity for rehydration. Similarly, post-embryonic development time will be longer, and emergence rate will be reduced as the quiescent period progresses due to smaller lipids reserves in the embryos. Meanwhile, since genetic mechanisms modulate the sexual proportions of mosquitoes, there will be no change in the proportion of males and females related to quiescence time. Finally, fecundity will be decreased as quiescence time increases, since low energy reserves result in smaller female body sizes, reducing their ability to ingest a blood meal and, consequently, egg production. In contrast, the fertility of females originating from quiescent eggs would not be affected because the eggs of these females were not exposed to the quiescence process.

## 2. Materials and Methods

### 2.1. Mosquito Strains

*A. aegypti* populations from two Brazilian municipalities 501 km apart from each other (Recife, 08°03′03″ S and 34°56′54″ W; Aracaju, 10°54′40″ S and 37°04′18″ W) were studied in this work. Both cities are dengue fever, chikungunya fever, and Zika virus transmission areas, and are characterized as Am Tropical monsoon climates based on the Köppen–Geiger climatic classification system, where the temperature of the coldest month is >18 °C and the precipitation on driest month is >100 mm [36].

Recife (8 m above sea level masl) is one of the oldest cities in Brazil and is considered one of the ten most-populous municipalities in the country. The city presents an average temperature of 25.9 °C and average rainfall of 1800 mm throughout the year [37]. Its estimated population in 2017 was 1,633,697 inhabitants [38]. In contrast, Aracaju (4 masl) is one of the least populated capital cities (650,106 people) [38] in Brazil. On average, annual temperatures of 26 °C and rainfall of 1590 mm are registered [39].

### 2.2. Parental Generation (PG)

The *A. aegypti* parental generation (PG) was obtained from eggs collected in urban areas, using oviposition traps (ovitraps) made of 500 mL black plastic pots, 15 × 12 cm. The oviposition trap contained water and a 15 × 5 cm strip of a wooden pallet as an oviposition substrate. In the laboratory, paddles containing eggs were placed in plastic trays (40 × 27 × 7 cm) covered with a mesh and left for drying for three days to complete embryonic development, at 26 ± 1 °C, 80 ± 5% RH and photoperiod of 12:12 [light:dark] h. After this period, eggs were transferred to a new plastic tray, filled with filtered water to allow larvae to hatch. Larvae were fed with fish food (Tetra^®^ Marine XL Granules, Melle, Germany) ad libitum until pupation. Pupae were transferred to plastic cups and placed inside breeding cages for adult emergence. Due to the presence of other *Aedes* species in both cities, a screening method based on the taxonomic key of Consoli and Lourenço-de-Oliveira [40] was used to identify adults of *A. aegypti* during the formation of the PG. After selection, mosquitoes were fed 10% sucrose solution ad libitum as a carbohydrate source. After sugar deprivation for 24–48 h, females were fed defibrinated and sterile sheep blood (EBE-FARMA Biológica e Agropecuária LTDA, Rio de Janeiro, Brazil) until full engorgement to stimulate ovogenesis. Three days after the blood meal, 50 mL plastic cups containing sterilized cone filter paper filled with 10 mL of water were placed inside the cages for oviposition for three days and exchanged every 24 h.

### 2.3. Quiescent Eggs

Eggs from F1–F3 generations were divided into groups and stored in plastic containers covered with a mesh for 10, 40, 70, 100, 130 and 160 days before use. The storage periods were chosen based on previous observations made in our laboratory [41]. From this, egg groups were formed for viability analyzes with a 30-day interval between groups. Eggs of 10 days old were used as a baseline (control) for the experiments. Although these eggs were in the beginning of the quiescent period, in our previous tests, hatching rate did not significantly differ from three days old eggs (freshly embryonated eggs) (mean of 49.2 ± 12.11; Kruskal–Wallis with Student–Newman–Keuls test a posteriori, *p* ≤ 0.05). Ten replicates for each group were used totalizing approximately 1000 eggs for each population and stored period.

### 2.4. Effect of Quiescence on Different Biological Parameters of A. aegypti

#### 2.4.1. Egg Viability

For each quiescence condition (10, 40, 70, 100, 130 and 160 days), the total number of larvae hatched following immersion of eggs in water was recorded after seven days (maximum period of larval hatching observed in previous tests). Egg viability was measured as the percentage mean of total egg hatching in each group.

#### 2.4.2. Initial Hatching Time

The presence of the first larvae was recorded in hourly observations for each quiescence time to evaluate whether older eggs would require a longer water immersion time for larval hatching. Observations performed before an hour resulted in no larval hatching.

#### 2.4.3. Post-Embryonic Development Time

A maximum sample of 10% of the total number of larvae obtained in each replicate in the above experiments was used to investigate whether the time for larvae to reach adulthood would increase according to a longer quiescence period. This value was determined to keep the same proportion for each replicate, thus avoiding bias due to sampling errors. Larvae (up to 8 h after hatching) were individualized in plastic cups (50 mL capacity) with 40 mL of water and fed with fish food, in proportion to 2 mg/larvae on alternate days. Upon reaching the pupa stage, the cups containing individuals were transferred to cages (transparent container with 15 cm diameter and 9 cm in height, and 1000 mL volume) separately for adult emergence. The period required to complete the life cycle (L1 to adult), in days, was registered.

#### 2.4.4. Emergence Rate and Sexual Proportion

After their emergence, males and females were quantified, and the male/female proportion was determined. Adults were left for mating in adult cages (40 × 40 cm) and used for fecundity and fertility trials described below. After females received a blood meal (as previously described), mosquitoes were fed on sugar solution ad libitum.

#### 2.4.5. Fecundity and Fertility

Immediately after a blood meal, engorged females were individualized into transparent plastic receptacles (1000 mL) fitted with a breeding site (50 mL plastic cup containing filter paper soaked in water). A cotton swab moistened with a 10% sucrose solution was offered ad libitum. After a week, breeding sites were removed, dried at room temperature, and eggs counted under a stereomicroscope using 10× magnification (LEICA Microsystems, model S8 AP0, Wetzlar, Germany). The total number of eggs laid by a female can be altered throughout the gonotrophic cycle (GC) [15]. Thus, for this study, the number of eggs deposited at the end of the first GC was used as an estimate of fecundity. Fertility assays were performed using eggs from the fecundity trials. Three days after the removal of the oviposition sites, larvae were hatched by submerging the eggs in water, and the eclosion rate was evaluated after seven days.

#### 2.4.6. Adult Size

To estimate adult size, left wings were detached and the distance from the axillary incision to the apical margin, excluding the fringe of scales, was measured using a stereomicroscope with a coupled camera [42,43] using a magnification of 25× (LEICA Microsystems, model S8 AP0, Wetzlar, Germany).

### 2.5. Statistical Analyses

Lilliefors and Bartlett tests were used to verify the normality of distribution and homoscedasticity of all data. All analyses were performed using BioEstat software, version 5.3 [44], with values of *p* ≤ 0.05 indicating statistical significance. To analyze whether the quiescence period affects viability, initial hatching time, post-embryonic development time, adult emergence rate, size, fecundity, and fertility we used the Kruskal–Wallis test followed by the Student–Newman–Keuls (SNK) multiple comparison tests. Differences in sexual proportion were determined using the Qui-Square Adhesion test for samples with the same expected proportions of 50:50% (males:females) (with a correction of Yates). Lastly, the Mann–Whitney U-test for independent samples and unequal sizes was used to calculate *p*-values and determine the significance between populations of mosquitoes. Results were expressed as average followed by confidence interval 95% (CI 95%) and range. The graphical representation of the data was made using box plots and bar graphics were produced in the Microsoft Office Excel software, version 2016.

## 3. Results

### 3.1. Egg Viability

**Figure 1:**
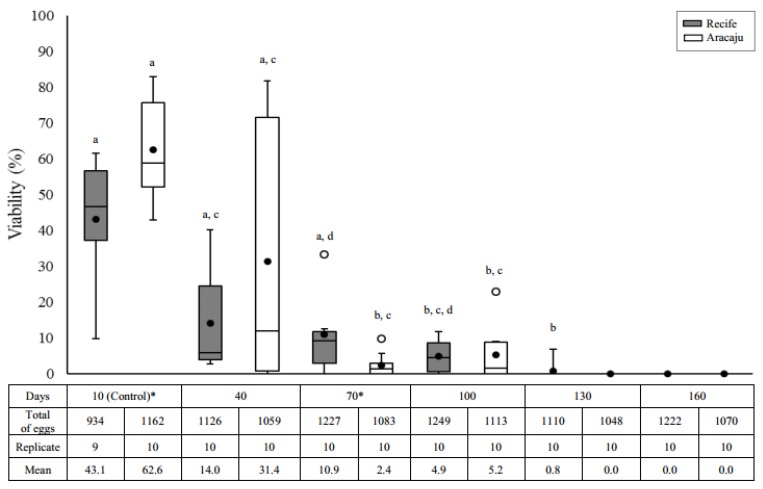
Eclosion rate (%) as a function of the quiescent period in two populations of *Aedes aegypti*—Recife (Gray) and Aracaju (White). Replicates consist of approximately 100 eggs. The size of the boxes indicates the distance between the first (lower) and third (upper) quartiles, the central mark among them shows the median and the closed circle the mean; the bars indicates variability outside quartiles and outliers are plotted as empty circles. The letters indicate comparisons between the different quiescent periods within each population (Kruskal–Wallis with Student–Newman–Keuls test a posteriori). Different letters indicate significant differences at *p* ≤ 0.05. Asterisks indicate significative difference interpopulation in the same period of quiescence (Mann–Whitney U-test, *p* ≤ 0.05).

Overall, egg viability significantly decreased in both populations as time progressed (Recife, H = 35.0000; df = 5; *p* = 0.0000 and Aracaju, H = 32.7493; df = 5; *p* = 0.0000), although a differential response in the reduction pattern of the eclosion rate had been observed. Maximum egg viability lasted a month longer in eggs from Recife (130 days; 0.8%) compared to the Aracaju population (100 days; 5.2%) (Figure 1). A significant reduction in egg viability was recorded after 40 and 70 days of quiescence in the Aracaju (*p* = 0.0021) and Recife (*p* = 0.0077) populations, respectively. A wide variation in hatching rate was observed, being more expressive at ten days in Recife (9.8–61.5 days) and 40 days in Aracaju (0–81.8 days). Significant differences between populations were observed in egg storage for 10 (U = 20; *p* = 0.0412) and 70 days (U = 23.5; *p* = 0.0452). In comparison with the Recife population, eggs from Aracaju showed a 70% higher eclosion rate at ten days and a 20% reduction after 70 days of storage.

### 3.2. Initial Hatching Time

**Figure 2:**
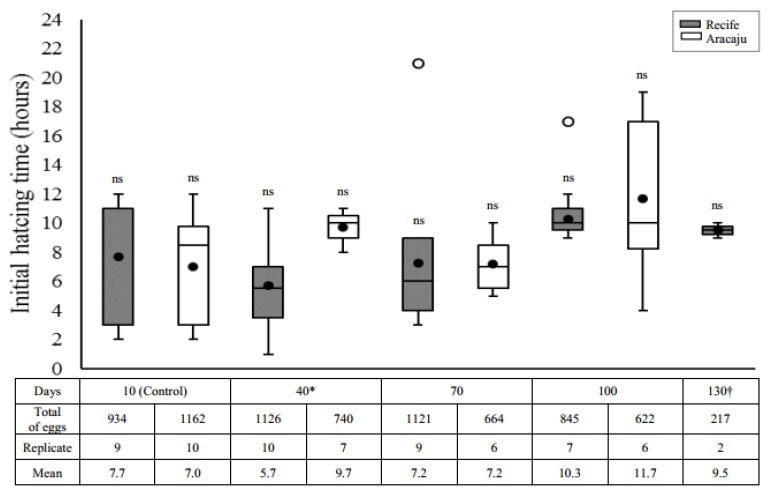
Initial hatching time (hours) of *Aedes aegypti* larvae in different quiescent periods, in two mosquitoes populations—Recife (gray) and Aracaju (white). The number of replicates was obtained according to the viability test, each them consisting of approximately 100 eggs. Values are represented as average hatching time (closed circle); the size of the boxes indicates the distance between the first (lower) and third (upper) quartiles, the central mark among them shows the median; the bars indicates variability outside quartiles and outliers are plotted with empty circles. No significant difference (ns) was found between the quiescent period analyzed within each mosquito population separately (Kruskal–Wallis with Student–Newman–Keuls test a posteriori, *p* ≤ 0.05). Asterisks indicate significative difference interpopulation in the same period of quiescence (Mann–Whitney U-test, *p* ≤ 0.05). No hatching was observed for the Aracaju mosquito population from 130 days, and no hatching was observed with 160 days of quiescence for both populations (see viability tests, Figure 1).

The mean time (hour) required for the first larvae to hatch after all quiescent periods is presented in Figure 2. Neither quiescent eggs from Recife nor from Aracaju required extended immersion times for the first hatching due to their quiescence period (Recife, H = 6.1401; df = 4; *p* = 0.1889 and Aracaju, H = 5.0897; df = 3; *p* = 0.1653). However, we noted a differential hatching pattern between the different populations. Under the quiescent conditions used in this work, most larvae in both populations hatched after seven h (>55%). Even so, eggs from Recife showed a greater range of hatching time (1–21 h) compared with eggs from Aracaju (2–19 h). The greatest difference between the two populations occurred in eggs stored for 40 days, with a significantly shorter mean hatching time in Recife population (5.7 ± 2.13 h) compared to Aracaju (9.7 ± 1.16 h) (U = 8; *p* = 0.0084).

### 3.3. Post-Embryonic Development Time

**Figure 3:**
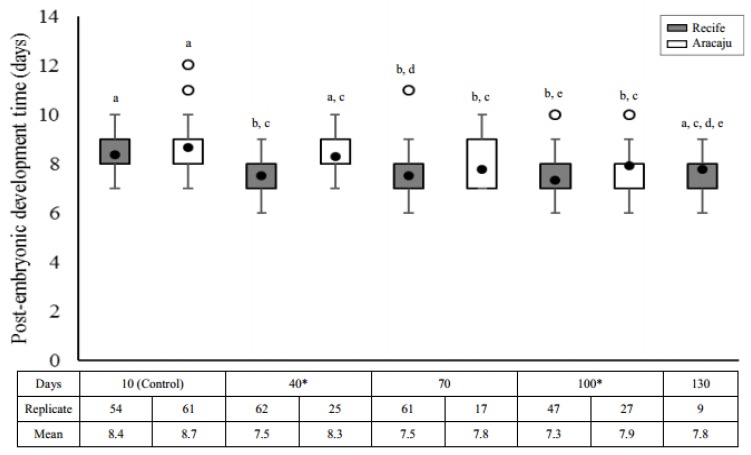
Post-embryonic development time (days) for immature *Aedes aegypti* obtained from eggs with different quiescence periods in two mosquitoes populations—Recife (gray) and Aracaju (white). Replicates represent the number of larvae obtained in the viability tests (approximately 10% of total hatchling). Values are represented as average post-embryonic development time (closed circle); the size of the boxes indicates the distance between the first (lower) and third (upper) quartiles, while the central mark among them shows the median; the bars indicates variability outside quartiles and outliers are plotted with empty circles. The letters indicate comparisons between the different quiescent periods within each population (Kruskal–Wallis with Student–Newman–Keuls test a posteriori). Different letters indicate significant differences at *p* ≤ 0.05. Asterisks indicate significative difference interpopulational for comparisons of values of the same period of quiescence (Mann–Whitney test, *p* ≤ 0.05).

In both populations, larvae from older eggs reached adulthood in a shorter time compared to controls (Recife, H = 47.2926; df = 4; *p* = 0.0000 and Aracaju, H = 16.1884; df = 3; *p* = 0.001). Overall, the cost of quiescence to immature development was lower in Recife (shorter development time) compared to the Aracaju population (longer development time) (Figure 3) with a significant reduction at 40 d (U = 368; *p* = 0.0001) and 100 d (U = 406; *p* = 0.0182). In Recife, larvae from the control group averaged 8.4 ± 0.18 d to reach adulthood, a period significantly higher compared to intermediate quiescent periods (40 days, *p* < 0.0001; 70 days, *p* < 0.0001 and 100 days, *p* < 0.0001), even including outliers at 70 and 100 days. No difference was observed between day 10 and 130 (*p* = 0.1046). However, the difference in sample sizes may represent a bias in this result. Meanwhile, shorter larval development times were observed for individuals from the Aracaju population eggs after 40 days of quiescence (70 days, *p* < 0.0013 and 100 days, *p* < 0.0057) when compared with control eggs.

### 3.4. Emergence Rate and Sexual Proportion

Overall, the proportion of adults emerging from pupae was dependent on the studied population and the quiescent period. The effect of the quiescent period was clearly seen in the mosquitoes from Recife (H = 9.9393; df = 4; *p* = 0.0415), but not in mosquitoes from Aracaju (H = 5.0264; df = 3; *p* = 0.1699). Also, although the proportion of viable eggs had decreased during the quiescent periods in both populations (Figure 1), proportionately more adults were obtained from older eggs in the Recife population. For instance, from a total of 94 L1 larvae (10 days of quiescence) individualized in a viability trial (shown above), 54 emerged as adults (57.5%). This percentage increased to 86.8% (*n* = 75) after 40 days of quiescence, reaching 100% (*n* = 9) at 130 days (Table 1). Conversely, the effect of the quiescence period on adult emergence showed no clear pattern for the Aracaju population. When the samples from each quiescent period were compared between the studied populations, significant differences in the emergence rate was observed in eggs after 40 days of quiescence (U = 6; *p* = 0.0047). In this situation, the total number of adults was 2.5 times greater in the Recife population (86.8%) than Aracaju (34.2%) (Table 1).

**Figure 4:**
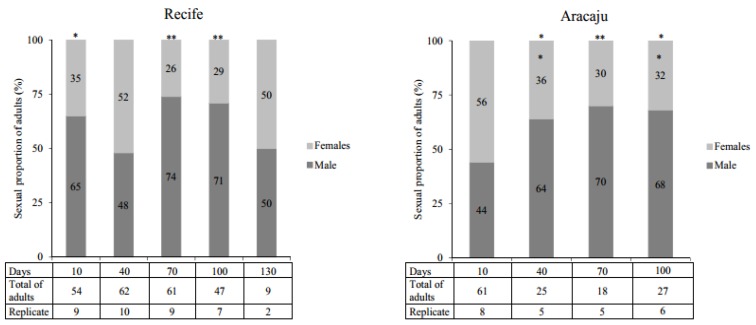
The proportion of *Aedes aegypti* adults (%) observed from eggs with different periods of quiescence. Replicates indicates the number of samples that presented emergence of adults from the viability tests. Asterisks identify within each population the periods of quiescence with significantly different deviations from the expected 50:50% (males: females) sexual proportion (Qui-Square test; * *p* < 0.005, ** *p* < 0.0001).

In general, quiescence did not affect the ratio of females and males within the timelines of both *A. aegypti* populations studied. However, when the expected sexual proportion of 50:50% was evaluated, significant differences were observed, particularly, at 70 and 100 days (Figure 4). No difference between populations was recorded.

### 3.5. Fecundity and Fertility

Fecundity was not affected by the quiescence period in either *A. aegypti* population (Recife, H = 4.5183; df = 3; *p* = 0.2107 and Aracaju, H = 1.2361; df = 3; *p* = 0.7444). Independent of the quiescence period, females from the Recife population (*n* = 77 females) laid an average of 46.3 ± 6.60 eggs/female (range 1–132,), while those of Aracaju (*n* = 53) presented a mean rate of 44.2 ± 8.05 (range of 3–123). In both populations, maximum and minimum fecundity rates were observed at 70 (Recife, 56.2 ± 10.00 and Aracaju, 54.8 ± 32.88) and 100 days (Recife, 40.3 ± 24.98 and Aracaju, 29.2 ± 22.19), respectively. No significant difference was found when comparing each period of quiescence between populations.

Fertility was also similar among groups and populations (Recife, H = 6.8691; df = 3; *p* = 0.0762 and Aracaju, H = 0.3967; df = 3; *p* = 0.9409). Approximately, 28% ± 4.44 (*n* = 2159 eggs) and 27.9% ± 5.81 (*n* = 1478) of all eggs hatched from Recife and Aracaju, respectively. The first population presented maximum fertility rates when females were emerged from eggs after 70 days (34.7 ± 6.20), while for the second population the optimal time was ten days (28.6 ± 8.41). Conversely, the minimum larval hatching rates were recorded for females from young eggs (10 days) in Recife and old eggs (100 days) in Aracaju. Significant differences between *A. aegypti* populations were otherwise not identified.

### 3.6. Size

Male and female body sizes were not affected by quiescence. However, females from the Recife population were larger than those from Aracaju, particularly those that emerged after 70 (U = 34.5; *p* = 0.0127) or 100 days (U = 53; *p* = 0.0136) quiescent eggs. Intrapopulation and interpopulation differences in male and female sizes are summarized in Table 2. Overall, females originating from quiescent eggs in the Recife population averaged 2.7 ± 0.03 cm (range 2.3–2.9 cm) and males 2.1 ± 0.06 cm (range 1.2–2.8 cm). The average wing size of females from eggs from the Aracaju population was 2.6 ± 0.05 cm (range 2.0–2.9 cm) and 2.1 ± 0.05 cm for males (1.8–2.7 cm).

## 4. Discussion

Studying different populations of *A. aegypti* we found a negative quiescent cost to egg viability, and sexual rate, which was dependent on the population studied and egg age (quiescence period). However, important parameters for population dynamics and vectorial capacities such as fecundity, fertility, and mosquito size were not affected by quiescence. In agreement with previous studies on egg quiescence in *A. aegypti* indicating differential egg viability [12,17,21,22,45,46,47], in our study, the *A. aegypti* population from Recife proved to be more resistant to desiccation (130 days, with high egg viability until three months of quiescence), than mosquitoes from the Aracaju population (100 days, with a high hatching rate up to 40 days of quiescence). Even so, the overall quiescence was shorter compared with other similar studies in which maximum larvae hatching was recorded from 150 days up to more than a year [12,22,46,47,48].

The reasons for the observed differences in mosquito egg resistance to desiccation are complex and involve a range of different intrinsic and extrinsic egg characteristics [17,19,21,22,49,50]. Variations in abiotic factors, particularly, those related to climate events (temperature and humidity), associated with egg water loss [51], and geographic characteristics (altitude), or causing variations in oxygen availability [52,53] have been associated with egg resistance to desiccation. Variations in rainfall (Recife 1804 mm; Aracaju 1409 mm), but not in temperature (Recife 25.8 °C and Aracaju 25.6 °C) observed between the two cities indicate that humidity is a more important factor in egg desiccation than the temperature for these populations. Also, additionally, Recife, at 7 m above sea level, has a slightly higher altitude compared with Recife, at 4 m above sea level. Egg viability in both populations was shorter compared with mosquito egg viability from Manaus (north Brazil), which has a higher average environmental humidity due to rainfall throughout the year and an altitude of 92 m [38]. Elevation of altitude decreases the oxygen available for absorption by individuals [52,53], and lower quantities of dissolved oxygen in breeding sites may affect egg hatching [54,55,56]. Although our data originates from laboratory observations, the use of eggs from the F1–F3 generations implies that many characteristics intrinsic to each population remain in the analyzed samples.

In addition to the variation of the maximum egg quiescence period, our results also demonstrate the cost of quiescence on the rate of emergence and sexual proportion in individuals of both populations, but a gain in the duration of post-embryonic development. All these biological parameters were modified across the time of quiescence for both mosquito populations—Recife and Aracaju. Relatively few studies have specifically addressed the effects of quiescence on the time of larval development after hatching, and divergent results have been reported. According to Silva and Silva [12], egg quiescence has no cost for development, ranging from 7.7 to 10.3 days. Conversely, Perez and Noriega [32] showed a significant increase in the duration of larval development in individuals from older eggs (7.33 days) compared with younger eggs (5.95 days), attributing such differences to a nutritional and physiological cost of quiescence time on larval development related to decreased lipids. Contrasting with these studies, we found that larvae from both populations developed 16–24 h faster in older compared with younger eggs whose averages were 8.4 ± 0.18 (range of 7–10) and 8.7 ± 0.26 (7–12) days to reach adulthood in Recife and Aracaju, respectively. These results may indicate an adaptive response for recolonization after starvation. These changes could be an adaptation in response to selection that favors the rapid production of mosquitoes. Intrinsic differences of larval homeostatic control of metabolite levels and greater efficiency in decreasing the rates of energy depletion may improve survival of larvae from older eggs, as observed for *Drosophila* [57,58].

Also, such efforts seem to have no negative effects on adult formation since the time of egg quiescence increased the number of adults in the Recife population but had no influence on the Aracaju population. Considering that adult formation is an important aspect in population density, the differential impact of quiescence in adult formation between the two populations implicate a distinct contribution to population dynamics and a lesser extent, to vectorial capacity. Thus, in the wild, mosquitoes from the Recife population could achieve a 100% increase of emergent adults after 130 days, while those from the Aracaju population would achieve a smaller proportionate increase (73.6%) but in a shorter period (100 days).

Overall, in our study, the proportion of males in both populations was greater than females in most of the quiescent periods. Despite this, no significant variations in gender proportions were observed over time. There was a sexual disproportion, particularly after 70 and 100 days quiescence, resulting in an excess number of males. These results are consistent with previous reports [22,59]. Recent research has confirmed that sex determination in *A. aegypti* does not involve an XY chromosome but an endogenous meiotic driver system that can cause sexual proportion distortion with a predominance of males over females [60,61].

Contrary to our hypothesis that older eggs would require more time for rehydration and consequently a longer time to larval hatching compared with the younger eggs, the quiescence period did not affect the initial hatching time, which remained between 5.7 h and 11.7 h in both populations. In general, younger eggs tend to hatch between 5 and 20 min after submersion, whereas older eggs, conditioned for months may require more than more 24 h for the appearance of larvae [22]. Although considered long, the times observed in our study were also mentioned by Christopher [22] who noticed an irregular time for hatching that varied from 1 to 5 days or more in floating eggs before submersion in water.

The ability of *A. aegypti* eggs to resist desiccation has been assumed to contribute to the invasion and spread of this mosquito worldwide and, consequently, the arbovirus transmitted by this species [17,18,19,21,24]. The differential capacity of eggs to resist desiccation among populations implies a differential contribution to these events, also affecting the success of control measures. Over the last few years, huge strides have been made in reducing *A. aegypti* populations, with the creation of maps of distribution and maximum risk of their establishment [2,62]. However, lack of up-to-date information regarding the factors affecting the distribution of *Aedes* species hampers surveillance and control. The results presented here revealed that different populations of *A. aegypti* have distinct phenotypes in response to quiescence, by modifying parameters of viability, post-embryonic development time, adult emergence rate, and sexual proportion. As a consequence, quiescence may impact measures of control, population dynamics, vector capacity, and competence by re-infestation of previously treated areas and an increase in the population density associated with a short life cycle. Additionally, our results provide information on the behavior of these populations for improvement of surveillance and control purposes, additional knowledge about egg resistance to desiccation, and provide support for new research aimed at fitness, geographic expansion, and transmission of arboviruses by *A. aegypti*.

## 5. Conclusions

In summary, our data show that out of eight parameters evaluated, four were affected by the quiescence (viability, post-embryonic development time, emergence rate and sexual proportion). These findings reinforce the idea that quiescent eggs are a public health problem, directly or indirectly affecting control programs, population dynamics, vector capacity, and competence of mosquitoes. Additionally, it is important for future research aimed at answering questions about the biology of *A. aegypti*, take into account the quiescence period of eggs since this factor can significantly influence these characteristics.

## Figures and Tables

**Table 1:** Emergence rate means ± CI 95% (range of values) from *Aedes aegypti* eggs of the Recife and Aracaju populations according to quiescence periods.

Period of Quiescence (Day)	Population		Mann–Whitney U-Test	*p*-Value
	Recife	Aracaju		
10 days	57.5 ± 22.76 (10.0–100) ^a^ *n* = 94	52.8 ± 23.13 (0–91.7) ^a^ *n* = 117	41.5	0.7751
40 days	86.8 ± 9.74 (61.5–100) ^b,c^ *n* = 75	34.2 ± 30.48 (0–90) ^a^ *n* = 66	6.0	0.0047
70 days	74.5 ± 15.40 (40.0–100) ^a,c^ *n* = 88	61.7 ± 35.37 (0–100) ^a^ *n* = 26	18.5	0.3165
100 days	84.5 ± 12.29 (66.7–100) ^a,c^ *n* = 58	73.6 ± 30.14 (22.2–100) ^a^ *n* = 42	17.0	0.5700
130 days	100 ± 0.00 (100–100) ^b,c^ *n* = 9	No emergence	NA	NA
160 days	No emergence	No emergence	NA	NA

*n* = Total number of L1 larvae that were individualized; Small letters indicate comparisons of values (Kruskal–Wallis with Student–Newman–Keuls test a posteriori; *p* ≤ 0.05) in the same column. Values identified by the same letter types are not significantly different; The Mann–Whitney test (*p* ≤ 0.05) was used to assess interpopulational differences between the emergence rate means; NA = Not applicable.

**Table 2:** Average size ± CI 95% (range of values) of adult males and females from *Aedes aegypti* of the Recife and Aracaju populations according to quiescence periods.

Period of Quiescence	Population				Mann–Whitney Test		*p*-Value	
	Recife		Aracaju					
	Males	Females	Males	Females	Males	Females	Males	Females
10 days	2.1 ± 0.13 (1.6–2.7) ^a,A^ *n* = 15	2.7 ± 0.09 (2.3–2.9) ^a,B^ *n* = 15	2.1 ± 0.06 (2.0–2.3) ^a,A^ *n* = 15	2.7 ± 0.09 (2.3–2.9) ^a,B^ *n* = 15	99.0	94.5	0.5755	0.4553
40 days	2.2 ± 0.11 (2.0–2.5) ^a,A^ *n* = 15	2.6 ± 0.09 (2.4–2.9) ^a,B^ *n* = 15	2.2 ± 0.10 (1.9–2.5) ^a,A^ *n* = 15	2.6 ± 0.17 (2.0–2.9) ^a,B^ *n* = 10	100.5	69.5	0.6187	0.7603
70 days	2.1 ± 0.10 (1.9–2.6) ^a,A^ *n* = 15	2.7 ± 0.05 (2.5–2.9) ^a,A^ *n* = 15	2.2 ± 0.33 (1.9–2.7) ^a,A^ *n* = 06	2.5 ± 0.12 (2.2–2.8) ^a,B^ *n* = 11	37.0	34.5	0.5334	0.0127
100 days	2.2 ± 0.15 (1.9–2.8) ^a,A^ *n* = 15	2.8 ± 0.04 (2.7–2.9) ^a,B^ *n* = 15	2.1 ± 0.11 (1.8–2.7) ^a,A^ *n* = 15	2.6 ± 0.08 (2.3–2.8) ^a,B^ *n* = 15	51.0	53.0	0.9158	0.0136
130 days	2.0 ± 0.36 (1.2–2.4) ^a,A^ *n* = 07	2.7 ± 0.52 (2.7–2.8) ^a,B^ *n* = 02	NA	NA	NA	NA	NA	NA

*n* = Total number of adult mosquitoes; Small letters indicate comparisons of values (Kruskal–Wallis with Student–Newman–Keuls test a posteriori; *p* ≤ 0.05) in the same column, whereas capital letters indicate comparisons of values (Kruskal–Wallis with Student–Newman–Keuls test a posteriori; *p* ≤ 0.05) in the same row. Values identified by the same letter types are not significantly different (*p* > 0.05); The Mann–Whitney test (*p* ≤ 0.05) was used to assess interpopulational differences between males and females; NA = Not applicable.
